# Bevacizumab and Combination Chemotherapy in rectal cancer Until Surgery (BACCHUS): a phase II, multicentre, open-label, randomised study of neoadjuvant chemotherapy alone in patients with high-risk cancer of the rectum

**DOI:** 10.1186/s12885-015-1764-1

**Published:** 2015-10-23

**Authors:** R. Glynne-Jones, N. Hava, V. Goh, S. Bosompem, J. Bridgewater, I. Chau, A. Gaya, H. Wasan, B. Moran, L. Melcher, A. MacDonald, M. Osborne, S. Beare, M. Jitlal, A. Lopes, M. Hall, N. West, P. Quirke, Wai-Lup Wong, M. Harrison, for the Bacchus investigators

**Affiliations:** 1 Radiotherapy Department, Mount Vernon Centre for Cancer Treatment, Mount Vernon Hospital, Northwood, UK; 2 Cancer Research UK & University College London Cancer Trials Centre, London, UK; 3 Division of Imaging Sciences & Biomedical Engineering, Kings College London, London, Department of Radiology, Guy’s and St Thomas’ Hospitals NHS Foundation Trust, London, SE1 7EH UK; 4 Pharmacy, Mount Vernon Centre for Cancer Treatment, Mount Vernon Hospital, Northwood, UK; 5 University College, London Cancer Institute, 72 Huntley St., London, WC1E 6AA UK; 6 Department of Medical Oncology, Royal Marsden Hospital, London & Surrey, UK; 7 Department of Cancer Medicine, Hammersmith Hospital, Imperial College Healthcare NHS Trust, London, UK; 8 Department of Surgery, Hampshire Hospitals Foundation Trust, Basingstoke, Hampshire UK; 9 Radiotherapy Department, Beatson Oncology Centre, 1053 Great Western Rd, Glasgow G12 0YN, UK; 10 Radiotherapy Department, North Middlesex Hospital, Sterling Way, London N18 1QX, UK; 11 Radiotherapy Department, Royal Devon & Exeter Hospital, Barrack Rd, Exeter, Devon EX2 5DW UK; 12 Leeds Institute of Cancer and Pathology, School of Medicine, University of Leeds, Leeds, United Kingdom; 13 Department of Radiology, Paul Strickland Scanner Centre, Mount Vernon Centre for Cancer Treatment, Mount Vernon Hospital, Northwood, UK; 14 Radiotherapy Department, Guys and St Thomas’s Hospital, Westminster Bridge Road, London, SE1 7EH UK

**Keywords:** FOLFOXIRI, FOLFOX, Bevacizumab, Locally advanced rectal cancer, Total mesorectal excision, Resectable, Metastatic disease

## Abstract

**Background:**

In locally advanced rectal cancer (LARC) preoperative chemoradiation (CRT) is the standard of care, but the risk of local recurrence is low with good quality total mesorectal excision (TME), although many still develop metastatic disease. Current challenges in treating rectal cancer include the development of effective organ-preserving approaches and the prevention of subsequent metastatic disease.

Neoadjuvant systemic chemotherapy (NACT) alone may reduce local and systemic recurrences, and may be more effective than postoperative treatments which often have poor compliance. Investigation of intensified NACT is warranted to improve outcomes for patients with LARC. The objective is to evaluate feasibility and efficacy of a four-drug regimen containing bevacizumab prior to surgical resection.

**Methods/design:**

This is a multi-centre, randomized phase II trial. Eligible patients must have histologically confirmed LARC with distal part of the tumour 4–12 cm from anal verge, no metastases, and poor prognostic features on pelvic MRI. Sixty patients will be randomly assigned in a 1:1 ratio to receive folinic acid + flurourcil + oxaliplatin (FOLFOX) + bevacizumab (BVZ) or FOLFOX + irinotecan (FOLFOXIRI) + BVZ, given in 2 weekly cycles for up to 6 cycles prior to TME. Patients stop treatment if they fail to respond after 3 cycles (defined as ≥ 30 % decrease in Standardised Uptake Value (SUV) compared to baseline PET/CT).

The primary endpoint is pathological complete response rate. Secondary endpoints include objective response rate, MRI tumour regression grade, involved circumferential resection margin rate, T and N stage downstaging, progression-free survival, disease-free survival, overall survival, local control, 1-year colostomy rate, acute toxicity, compliance to chemotherapy.

**Discussion:**

In LARC, a neoadjuvant chemotherapy regimen - if feasible, effective and tolerable would be suitable for testing as the novel arm against the current standards of short course preoperative radiotherapy (SCPRT) and/or fluorouracil (5FU)-based CRT in a future randomised phase III trial.

**Trial registration:**

Clinical trial identifier BACCHUS: NCT01650428

## Background

In LARC, preoperative chemoradiation and radiotherapy have become accepted as the standard of care. Meta-analyses [1] and individual trials of SCPRT [2, 3] and CRT [4–7] have demonstrated that radiotherapy improves local control, but provides no impact on OS [2, 3, 8]. Radiotherapy is associated with significant late-effects [9], including an increased risk of second malignancies [10, 11].

Recent improvements in the quality of surgery, preoperative magnetic resonance imaging (MRI) and pathological reporting, now call into question the approach of treating all patients, clinically staged as T3, with radiotherapy or chemoradiation to prevent local recurrence. Rather factors that portend distant recurrence should be considered, including tumour location and the sub-classifcation of T3, nodal status and presence of extramural invasion. For carefully selected patients low rates of local recurrence can be achieved if good quality TME is performed even when patients receive no radiotherapy [12–14]. Metastatic disease, in contrast, is now the predominant cause of recurrence and death. It appears a commonly held belief that any systemic chemotherapy treatment, is likely to be more effective if administered before and not after radical surgery.

For colon cancer, systemic treatment is given postoperatively based on histopathogy of the surgical specimen. The concept of neoadjuvant chemotherapy (NACT) is being examined in primary colon cancer in the FOXTROT trial (ISRCTN 87163246.) with promising early results [15].

For rectal cancer staging MRI can identify patients at risk of local and/or systemic relapse preoperatively. In particular, extramural vascular invasion (EMVI) is easily identified on preoperative MRI, and predicts for systemic failure with good concordance between MRI diagnosed and eventual pathological confirmation of EMVI [16].

National Comprehensive Cancer Network (NCCN) CRC Guidelines recommend a 6-month postoperative course of adjuvant chemotherapy for patients with stage II/III rectal cancer following chemoradiation [17], although this is not evidence-based [18] and recent data suggests there is no benefit from adjuvant 5FU apart from reducing local recurrence [19]. Potential explanations include the difficulty in delivering systemic chemotherapy treatment following CRT and surgery [4–6, 20]. Consensus recommendations suggest that decisions regarding adjuvant chemotherapy in LARC should be dictated according to initial preoperative clinical stage [21, 22]. Neoadjuvant chemotherapy (NACT) has therefore been recommended as a priority of future research, to decrease the high metastases rate [23].

Previous studies suggest tolerability and compliance with chemotherapy in the neoadjuvant setting should be high [24–26]. In the Grupo Cáncer de Recto 3 study [27] NACT was delivered at full systemic doses to 94 % of patients. In the GEMCAD 0801 study, a 15 % pathological complete response (pCR) was achieved with capecitabine + oxaliplatin (XELOX) plus BVZ [28] without any radiotherapy.

The BACCHUS study examines whether intensive NACT can achieve a pCR rate in primary rectal cancer sufficient to warrant further investigation. Chemo-triplet schedules demonstrate high response rates [29]. The OLIVIA phase II study randomised 80 patients with unresectable colorectal cancer liver-only metastases [30] comparing FOLFOX plus BVZ with or without irinotecan and reported response rates of 61.5 and 80.5 % respectively with acceptable toxicity. These are the experimental arms in BACCHUS, which will allow evaluation of the potential benefit of BVZ in combination with modern effective-doublet and triplet chemotherapy regimens and omitting radiotherapy in LARC.

## Methods/design

### Study design

The BACCHUS trial is an investigator initiated, multicentre, open-label, prospective, randomized phase II study. All participants have to provide written informed consent, signed and personally dated, before inclusion in the trial. The trial EudraCT number is 2010-022754-17, and is registered on ClinicalTrials.gov (BACCHUS: NCT01650428).

### Trial organisation

The sponsor is University College London. Central coordination is financed by Cancer Research UK (CR UK) and carried out by the CR UK & University College London Cancer Trials Centre (UCL CTC). An independent data monitoring committee (IDMC) will monitor the conduct and safety of the trial. Participating sites are required to report all serious adverse events (SAE) as defined by the protocol to UCL CTC in line with applicable regulations.

### Ethics and informed consent

The final protocol was approved by Riverside l Research Ethics Committee (ref: 12/LO/1158). Appropriate approval from respective local ethics committee is required to join this trial. This study has been approved by the ethics committee of the following Hospitals or Universities: Barnet and Chase Farm Hospital, Blackpool Teaching Hospitals, East and North Herts Hospitals, NHS Greater Glasgow and Clyde Hospitals, Heatherwood and Wexham Park Hospitals, Hillingdon Hospitals, Imperial College Healthcare NHS Trust, North Middlesex University Hospital, The Royal Marsden Hospital, University College Hospital, London. This study is conducted in accordance with the most recent version of the Declaration of Helsinki and according to GCP. Written informed consent, signed and personally dated, is obtained from each patient before inclusion in the trial.

### Population

Patients with histologically confirmed adenocarcinoma of the rectum require specific tumour and patient criteria for inclusion. A staging MRI is mandated. The lists of inclusion and exclusion criteria are presented in Tables 1 and 2.

**Table 1:** Patient inclusion criteria

• Histologically confirmed diagnosis of adenocarcinoma of the rectum
• Distal part of the tumour within 4–12 cm of the anal verge
• No unequivocal evidence of established metastatic disease (on chest/abdominal/pelvis CT). Patients with equivocal lesions (as determined at MDT) are eligible
• MRI-evaluated-evaluated locally advanced tumour with the following:
• T3 tumours extending (≥4 mm), beyond the muscularis propria N0–N2
• Or tumours (involving or threatening the peritoneal surface) or presence of macroscopic extramural venous invasion (V2 disease)
• AND for tumours below the peritoneal reflection, the primary tumour or involved lymph node (on MRI) must be >1 mm from the mesorectal fascia
• Measurable disease (using RECIST criteria v1.1)
• WHO performance status 0 – 1
• In the opinion of the investigator:
▪ General condition considered suitable for radical pelvic surgery
▪ Candidate for systemic therapy with FOLFOX/FOLFOXIRI plus bevacizumab
▪ Adequate bone marrow, hepatic and renal function:
▪ Haemoglobin ≥80 g/L
▪ ANC ≥2 × 10^9^/L
▪ Platelet count ≥100 × 10^9^/L
▪ ALT or AST ≤1.5 × ULN (upper limit of normal)
▪ ALP ≤1.5 × ULN
▪ Total bilirubin ≤1.5 × ULN
▪ Serum creatinine ≤1.5 × ULN
▪ Creatinine clearance ≥50 mL/min using the Cockcroft–Gault formula (see Appendix 4). If the calculated GFR is below <50 ml/min, ^51^Cr-EDTA or ^99m^Tc-DTPA clearance test must be carried out demonstrating GFR is ≥50 ml/min
• INR ≤ 1.1
• Urine protein ≤1+ with dipstick or urine analysis.
▪ For proteinuria >1+ or urine protein/creatinine ratio ≥ 1.0, 24-h urine protein should be obtained and the level must be <2 g for eligibility
• No evidence of established or acute ischaemic heart disease on ECG and normal clinical cardiovascular assessment
• No known significant impairment of intestinal absorption
• At least 18 years of age, but not more than 75 years
• Willing and able to give informed consent, comply with treatment and follow up schedule

**Table 2:** Patient exclusion criteria

The following are exclusion criteria
1. Primary tumour or lymph node on MRI
• Extending <1mm from, or breaching the mesorectal fascia and therefore the circumferential resection margin,
• Disease outside of the mesorectal envelope (internal iliac/lateral pelvic lymph node),
2. Clinically significant cardiovascular or coronary disease ≤2 years before randomisation,
3. History of interstitial lung disease or evidence of interstitial lung disease on baseline chest CT scan
4. History of an arterial thromboembolic event during the previous 2 years
5. Evidence of bleeding problems or coagulopathy (Patients receiving warfarin/coumarin derived anticoagulants at full therapeutic doses are excluded, but prophylactic doses of 1 mg to prevent Hickman line clotting are eligible).
6. Significant and continuing rectal bleeding leading to a haemoglobin <80 g/L
7. Chronic use of aspirin (>325 mg/day) or clopidrogel (>75 mg/day) within 10 days of first planned study treatment;
8. Taking phenytoin or sorivudine or its chemically related analogues, such as brivudine
8. Patients requiring regular use of anti-diarrhoeal medication; (NB Patients with ileostomy will not be able to participate if they require regular use of anti-diarrhoeal agents)
9. Serious uncontrolled intercurrent illness including poorly controlled diabetes mellitus;
10. Metallic colonic or rectal stent *in situ*
11. Previous pelvic radiotherapy;
12. Previous treatment with another investigational agent within 30 days prior to randomisation;
13. Patients with a history of previous malignancy in the past 5 years, excepting basocellular or squamous cell skin cancer, or properly treated cervicouterine cancer *in situ;*
14. Known HIV, HBV or HCV infection;
15. Pregnant or lactating women or pre-menopausal women not using adequate contraception;
16. Current smoker, or clinically relevant history of drug or alcohol abuse;
17. Patients with any other condition or concurrent medical or psychiatric disease who, in the opinion of the investigator, are not eligible to enter the study.

Trial entry has been restricted to patients in whom MRI suggest primary tumour or lymph nodes do not extend to ≤1 mm from, or breach the circumferential resection margin (CRM)– since even with preoperative chemoradiation up to 30 % of these patients would have a positive CRM (≤1 mm) after TME. Eligibility is also confined to patients with MRI estimated penetration of the mucularis propria >1 mm and/or patients with cN2 predicted by MRI and extramural vascular invasion (EMVI), but T3 tumours must have a predicted ≥2 mm margin from the mesorectal fascia.

These criteria are likely to form a group of patients making up about 40 % of rectal cancers overall. Such patients have a 50 % 5 year survival [32] and a local recurrence rate of 6–10 % with surgery alone.

Trial entry has also been restricted to patients younger than 70 years with distal rectal tumours, 4–12 cm from the anal verge. Accurate clinical staging with MRI is more diffcult in the low rectum, at lower than 4 cm there is a 5–15 % risk of involved lateral pelvic lymph nodes, which are not resected at TME. A lack of evidence to suggest a benefit from oxaliplatin containing adjuvant chemotherapy for stage II colorectal cancer [34–36] and insufficient data to support a benefit from adjuvant chemotherapy in Stage III colorectal cancer in patients over 70 years has informed the decision to exclude patients over 70 years from this trial [33].

### Study objectives and endpoints

The primary objective of the BACCHUS study is to evaluate the efficacy of FOLFOXIRI + BVZ and FOLFOX + BVZ in terms of their ability to produce pCR. Secondary objectives include evaluation of the safety and tolerability of the two regimes and the feasibility of delivering them, as well assessment of additional measures of efficacy such as progression free and overall survival.

The primary endpoint is pathological complete response (pCR) at surgery; secondary endpoints include ORR, CRM negative (R0) resection rate, T and N stage downstaging, PFS, DFS, OS, local control, 1 year colostomy rate, adverse events, compliance with chemotherapy treatment, tumour regression grade (TRG), and tumour cell density (TCD).

Survival curves for DFS and OS will be plotted. Cumulative incidence of local recurrence will be computed accounting for death as competing risk. Differences in survival will be tested with the log-rank test. Hazard ratios and 95 % confidence intervals (CI) will be computed using Cox regression. A table will present the completion rate of the neo-adjuvant treatment, pCR frequency, patients with a R0 resection with 90 and 95 % CI. Frequency and percentages for toxicity will be presented according to the Common Terminology Criteria for Adverse Events (CTCAE) version 4.0. All proportions will be presented with 95 % CI.

### Randomisation and stratification

**Fig. 1:**
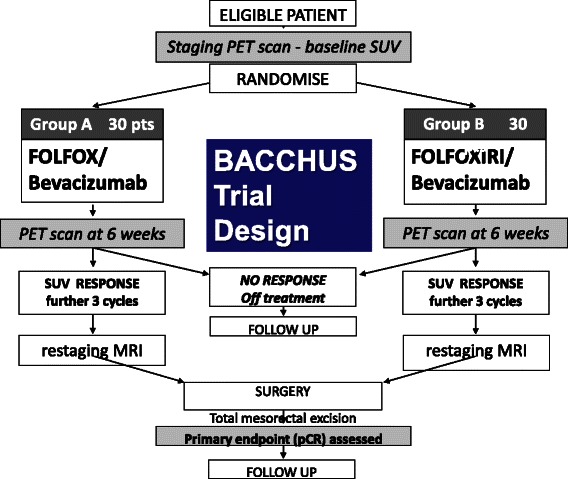
Treatment schedule

**Fig. 2:**
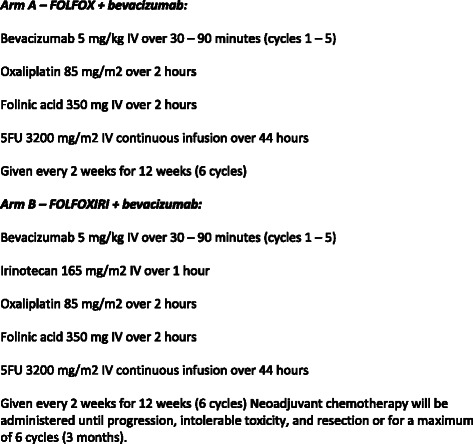
The BACCHUS trial. Patients will be randomised to one of two neoadjuvant chemotherapy regimens

Patient Randomisation will be performed centrally at the UCL trials centre. Eligible patients are randomly assigned to one of the two treatment arms in a 1:1 ratio and stratified according to treating centre, gender and presence or absence of EMVI. Treatment process and schedules for the BACCHUS trial are summarised in Figs. 1 and 2.

### Neoadjuvant Chemotherapy

In both arms, chemotherapy is delivered with bevacizumab. In total, 6 cycles of chemotherapy are prescribed preoperatively every 2 weeks (bevacizumab omitted during cycle 6). Adverse events are monitored from informed consent to 3 months after surgery and dose modification can be made according to specified protocol guidelines.

### Assessments/follow up

#### Response and resectabilty evaluation

Clinical response has not been shown to be a robust surrogate endpoint to predict outcome. However, patients will undergo response evaluation with MRI of the pelvis prior to cycle 4 and at the end of all treatment (prior to surgery) according to the Response Evaluation Criteria in Solid Tumours (RECIST 1.1) and additionally MRI-based TRG assessment is required [37]. An additional response evaluation according to standard uptake values (SUV) changes with PET/CT is mandated prior to cycle 4. Patients who do not respond will come off all trial treatment (allowing the investigator to proceed to whatever treatment is felt most appropraite ie surgery or SCPRT/CRT followed by surgery).

Tolerability to treatment is evaluated at each visit including physical examination, vital signs, WHO performance status, clinical laboratory profile, and adverse events, graded according to NCI-CTCAE v.4.03.

### Surgery and histopathology

Surgery should be performed 8–12 weeks after termination of chemotherapy, and a minimum of 8 weeks after the final dose of bevacizumab. Surgical dissection according to TME principles should not differ between the two trial groups and can be performed open or laparoscopically. Surgery may include anterior resection, abdominoperineal resection or a low Hartmann’s procedure.

Pathological evaluation of resected specimens will be according to guidelines included in the study protocol. The 5th edition of TNM will be used. In addition, circumferential resection margin (CRM) will be assessed and a margin of 1 mm or less considered positive. TRG will be presented as data categorised into five groups- TRG 0, TRG 1, TRG 2, TRG 3 and TRG 4 using the Dworak method. Also, the quality of the resected specimen will be evaluated with separate scoring for the mesorectum and the anal canal. Formalin fixed and paraffin embedded (FFPE) tumour tissue obtained at baseline will be evaluated for KRAS and BRA status and plasma /buffy coat collected at baseline and before the 2^nd^, 3rd and the 4th cycle (and also if the patient relapses), will be assessed for angiogenic markers (FFPE and serum) in the BACCHUS trial. Serum obtained at baseline, during preoperative treatment, postoperatively and at follow up will be evaluated for circulating tumour DNA.

### Adjuvant chemotherapy and follow-up

Patients can be treated with postoperative chemotherapy according to the local protocol of each participating centre. Patients will be followed up every 6 months for up to 42 months after randomisation, to document progression, recurrence and survival. Postoperative investigations/surveillance are performed according to local practice.

### Statistical considerations and sample size estimation

The primary endpoint for this trial is the pCR of the TME specimen. The proportion of patients in each arm who achieve a pCR will be presented, along with a 95 % CI. Within each group the achieved pCR rate will be compared to the historical rate achieved by radiotherapy alone (5 %). In the United Kingdom patients without a threat to the circumferential resection margin are likely to be treated with short course preoperative RT, and not chemoradiation. The study is powered on the assumption that a proportion of patients will have a pCR. It is well recognised that patients who have a complete clinical response (cCR) both on imaging and clinical examination will from time to time refuse surgery. For the purpose of this study, patients who have a sustained cCR at 12 months will be considered the same as a patient with a complete pathological response. Patients with a transient clinical response where subsequent relapse is observed within this 12 month period, will not.

Based on pCR with similar regimens prior to liver resection, and primary tumours responding better than metastases, we anticipate a pCR rate of 15–20 %. Compared to 5 % pCR rate historically for radiotherapy alone, a type I error α =0.05 and a type I I error β =0.8, 27 patients for the FOLFOX arm are required. The same number of patients is required for the FOLFOXIRI arm. Assuming 10 % of patients will be non-evaluable, 30 patients will be recruited to each arm (i.e. a total of 60 patients. NACT will be considered worth exploring further in a randomised phase III trial if at least 4/27 pCRs are observed. In the instance of more than 27 patients being assessed for pCR, the first 27 randomised patients per arm will be assessed. The study is not powered for a direct comparison between the two arms.

### Quality assurance/safety

Monitoring will be conducted centrally at UCL CTC and on-site monitoring will be scheduled if there is any evidence of non-compliance at site. An independent data safety monitoring committee (IDMC) meeting will be held periodically to review interim analysis, or as necessary to address any issues.

### Translational research

Analyses of both tumour tissue and plasma with tissue microarray, proteomics and genomics may generate increased knowledge of prognosis and prediction of response to chemotherapy in the BACCHUS trial. Hence, a schedule for collection of plasma and of fresh tissue for freezing, at different stages of treatment in each arm, is defined in the study protocol.

Tumour tissue and blood samples will be stored for future research. At surgical resection blocks of tumour and normal mucosa will be also be collected. In addition, H&E stained slides from the diagnostic biopsy and resection samples will be collected to undertake Tumour Cell Density and Tumour regression Grading.

Plasma and Peripheral Blood Leucocytes (PBL) samples will be collected at baseline and at the following timepoints during treatment :-Baseline (prior to starting treatment cycle 1), prior to starting treatment cycle 2, prior to starting treatment cycle 3, prior to starting treatment cycle 4 (preferably at radiological response assessment) and if patient relapses.

Conventional size–based radiological criteria using RECIST may not be the optimal method of assessing response to chemotherapy, especially with a regimen integrating bevacizumab) [37, 38]. Hence, imaging biomarkers will also be explored in terms of mri-based TRG and MRI diffusion weighted imaging [39]. Exploratory SPECT imaging using Tc99m-maraciclitide as the tracer in a subset of patients at baseline and post treatment will provide information regarding changes in angiogenesis with treatment.

## Discussion

### Why have we chosen to use bevacizumab?

Solid tumours are characterised by changes in structural architecture, which forms a barrier to uptake and penetration of cytotoxic drugs [40] and engenders hypoxia. Bevacizumab, a recombinant humanized monoclonal antibody against vascular endothelial growth factor (VEGF), increases response rates in metastatic colorectal cancer when combined with fluoropyrimidine-based regimen. When given neoadjuvantly, a VEGF inhibitor may act to prevent vessel formation and thus establishment of distant micrometastases.

In a non-randomised study comparing FOLFOXIRI and the four-drug intensive regimen combining FOLFOXIRI + BVZ in patients with liver metastases, 63 % of patients treated with FOLFOXIRI+ BVZ, versus 28 % treated with FOLFOXIRI/XELOXIRI alone, showed a histopathological response (*P* = 0.033) [41].

The BACCHUS study explores the use of bevaizumab in LARC with only potential loco-regional spread, which to some extent should limit evolutionary diversity in the tumour, and hopefully enhance response. All three adjuvant trials testing the role of bevacizumab; QUASAR, AVANT and CO8 excluded rectal cancer, because of the confounding issue of radiotherapy [42, 43] yet recent retrospective analysis of a cohort of 667 consecutive patients with metastatic colorectal cancer showed that patients treated with capecitabine, oxaliplatin and bevacizumab in whom the primary tumour originates in the rectum and/or sigmoid colon had better outcomes than patients with right-sided primary tumours [44]. Tumours in the distal colon and rectum also have higher expression of VEGF A (a hypothetical target of bevacizumab) than those in the proximal colon [45]. If there is an interaction between the location of the primary tumour and the effectiveness of antiangiogenic agents, future studies should stratify for the precise location of the primary tumour.

There is a consistently reported problem with delivery of, and compliance with chemotherapy following preoperative SCPRT or CRT and surgery. The EORTC 22921 trial showed compliance to postoperative adjuvant chemotherapy was very poor at 42.9 %. At least 25 % of patients in whom chemotherapy might be considered may not be sufficiently fit for treatment or decline [5, 6, 19]. The Chronicle trial highlighted this difficulty [46].

### Neoadjuvant chemotherapy for locally advanced rectal cancer

In locally advanced rectal cancer, the NSABP-R03 study employed a weekly schedule of 5FU and folinic acid for six weeks prior to definitive preoperative chemoradiation. A response rate of 44 % was achieved in the first 39 patients who completed all 6 cycles [7, 47]. Only 2 patients (5 %) progressed on this regimen. In a phase II study using neoadjuvant capecitabine and oxaliplatin, the clinical response rate was 88 % and no patient progressed radiologically [25]. Hence, anxieties that patients will progress on neoadjuvant chemotherapy appear unfounded.

The culture is now changing slowly away from the routine or blanket use of radiotherapy. The GEMCAD 0801 study achieved a 15 % pCR with XELOX + BVZ in [28] in a population very similar to those intended to be recruited into BACCHUS and without any radiotherapy. The Tribe study [31] showed a high clinical response rate in both arms - viz 53 % for FOLFOX + BVZ versus 65 % FOLFOXIRI + BVZ, with Grade 3 diarrhoea manageable at 9 and 19 % respectively.

Induction Bevacizumb and FOLFOXIRI has been shown to be a feasible regimen with acceptable toxicity (mainly neutropenia) in a multicentre study [49]. The ongoing Italian Trust study aims to treat 43 patients with LARC using FOLFOXIRI + BVZ followed by capecitabine based chemoradiation with bevacizumab. To date 23 patients have been randomised with a PCR of 38 %, and only 7 % surgical morbidity [52].

Our results should be better than the Tribe study since previous adjuvant chemotherapy impacted negatively on response in the FOLFOXIRI + BVZ arm. In BACCHUS, because the chemotherapy is neoadjuvant, patients will be chemotherapy naive. Since patients do not have metastatic disease, response rates for both arms should be even higher – probably in the region of 90 % since in the EXPERT C study XELOX and XELOX and cetuximab provided clinical response rates of 64 and 54 % respectively overall, and 71 % versus 51 % for patients expressing wild type KRAS [49].

### Limitations

The design of the BACCHUS trial has been criticised because, due to safety reasons and because patients over 70 years with stage II rectal cancer disease do not appear to benefit from *adjuvant* chemotherapy – particularly with oxaliplatin [33, 38]. Despite patients with rectal cancer across Europe having a median age at presentation of 71 years, an upper age limit of 70 years is mandated in BACCHUS because of these safety and futility concerns. The median age in most chemotherapy metastatic trials is 65 and the median age in most chemoradiation studies is 63 years [4–7, 50].

BACCHUS focuses on the efficacy and feasibility of preoperative FOLFOXIRI+ BVZ . The randomised design was chosen (albeit inevitably limited by the small number of patients) to compare efficacy in terms of pathological complete response and acute toxicity, in order to demonstrate the feasibility of avoiding radiation in this group of patients.

### Neoadjuvant chemotherapy without chemoradiation

Neo-adjuvant chemotherapy may achieve better access to malignant cells when the tumour has an intact blood supply, and offer better compliance to treatment [27] unlike an adjuvant approach which has failed to show any overall survival benefit in rectal cancer. Given neoadjuvantly, systemic doses of chemotherapy can be delivered at an earlier stage of disease rather than the delay of up to 18 weeks associated with standard CRT plus surgery. Two studies from the Memorial Sloan-Kettering Cancer Center (MSKCC) support the feasibility of neoadjuvant chemotherapy alone in rectal cancer [51, 52]. This feasibility study in patients with clinical stage II-III rectal cancer (but not T4 tumours) used FOLFOX + BVZ [52]. The R0 resection rate was the primary outcome. They reported a pCR in 8/29 patients (27 %). BACCHUS is a corroborative feasibility study but assessing more intensive chemotherapy in one arm. Based on the MSKCC results, a large multicentre Phase II/III study is currently accruing patients. In this CALGB PROSPECT/Allianz N1048 trial, patients are randomised to either 5FU-based chemoradiotherapy, surgery, and adjuvant FOLFOX chemotherapy, or the novel selective arm treating with 6 cycles of FOLFOX neoadjuvant chemotherapy and surgery alone. The primary endpoints of the Phase III components are time to local recurrence and disease-free survival.

Two small Japanese NACT studies have also demonstrated the feasibilty of this NACT approach and have included bevacizumab [53, 54]; there is a suggestion of increased surgical morbidity, but the rectal tumours were situated lower, on average 4.7 cm from anal verge, than those we hope to include in the BACCHUS study and surgery was performed earlier than specified in BACCHUS (3–8 versus 8–12 weeks) A higher dose of Bevacizumab was administered ie 7.5 mg/kg in these studies- in contrast to BACCHUS where the dose is 5 mg/Kg.

Finally the Olivia trial [30] used FOLFOXIRI and bevacizumab neoadjuvantly, in patients with mCRC deemed resectable and were offered surgery 5–7 weeks after their last bevacizumab dose and 3–5 weeks after their last chemotherapy cycle, a similar surgical timing of the 8–12 weeks mandated in BACCHUS.

The BACCHUS trial will therefore evaluate the efficacy of an intensive versus a standard first-line chemotherapy combination both with bevacizumab in patients with locally advanced/high risk rectal cancer to examine local control and long-term disease outcomes. Treatment duration is limited to a maximum of 3 months FOLFOXIRI + BVZ versus FOLFOX + BVZ.

PCR was chosen as the primary endpoint to confirm non-inferiority regarding the comparative efficacy with the standard chemoradiation option for these patients as this will then allow more confident treatment decisions to exclude radiotherapy for such patients in the future. Histopathological response is considered as a useful endpoint after chemotherapy for metastatic colorectal cancer (mcrc), representing a marker of sensitivity to preoperative treatments and a prognostic factor associated with longer survival [55, 56]. Although we are hoping in time to show that a neoadjuvant approach may influence overall survival, perhaps via biological/microenvironmental mechanisms surrounding micrometastases when a primary remains in situ, in contrast to adjuvant therapy,

In BACCHUS we are testing the feasibility of bevacizumab in a neoadjuvant setting where bleeding and perforation could prejudice the performance and quality of surgery. If the phase 2 passes tests of efficacy, safety and feasibility, we plan to develop a phase III study. Potential designs include, 3 months of neoadjuvant chemotherapy prior to surgery, followed by the option for a further 3 months of postoperative chemotherapy randomised against initial surgery followed by 6 months of postoperative adjuvant chemotherapy according to histology or alternatively against the current standard of SCPRT or chemoradiation.

## Conclusions

The BACCHUS trial will give further information about the feasibility, safety, tolerability and benefit of neoadjuvant FOLFOX or FOLFOXIRI + BVZ in this distinct disease setting of locally advanced but clearly resectable rectal cancer.

## Abbreviations

5-FU: 5-fluorouracil
5-y OSr: 5 year overall survival rate
AE: Adverse event
ALT: Alanin-aminotransferase
AST: Aspartat-aminotransferase
aPTT: Activated partial thromboplastin time
CA 19–9: Carbohydrate antigen 19–9
CEA: Carcinoembryonic antigen
CLM: Colorectal liver metastases
CRM: Circumferential resection margin
CRT: Chemoradiotherapy
CT: Computed tomography
DFS: Disease free survival
DNA: Deoxyribonucleic acid
ECOG-PS: Eastern cooperative oncology group – performance status
EGFR: Epidermal growth factor receptor
EMVI: Extramural Vascular Invasion
EORTC: European organisation for research and treatment of cancer
EudraCT: European Clinical Trials Database
FFS: Failure free survival
FFSR@18: Failure free survival rate at 18 months
FOLFIRI: 5-FU/LV and irinotecan
FOLFOX: 5-FU/LV and oxaliplatin
FOLFOXIRI: 5-FU/LV, oxaliplatin and irinotecan
G: Grade
G-CSF: Granulocyte colony-stimulating factor
HR: Hazard ratio
IFL: 5FU bolus and irinotecan regimen
INR: International Normalized Ratio
Iv: Intravenous
KRAS: Kirsten rat sarcoma viral oncogene homolog
LARC: Locally advanced Rectal Cancer
LV: Leucovorin
mCRC: Metastatic colorectal cancer
min: Minutes
MRI: Magnetic resonance imaging
N: Number of patients
NACT: neoadjuvant chemotherapy
NCI-CTCAE: National Cancer Institute common terminology criteria for adverse events
ORR: Overall response rate
OS: Overall survival
P: P-value
PBL: Peripheral Blood Leucocytes
pCR: Pathological complete response
PEI: Paul Ehrlich Institut
PFS: Progression free survival
PFSR@9: Progression free survival rate at 9 months
Po: Per os
RECIST: Response evaluation criteria in solid tumours
SAE: Severe adverse event
TCD: Tumour cell density
TRG: Tumour regression grade
TME: Total mesorectal excision
QoL: Quality of life
QLQ: Quality of life questionnaire
UICC: Union internationale contre le cancer
ULN: Upper limit of normal
V: Version
VEGF: Vascular endothelial growth factor
Vs: Versus
XELOX: Capecitabine and oxaliplatin
